# Trade-Offs of Plant Biomass by Precipitation Regulation Across the Sanjiangyuan Region of Qinghai–Tibet Plateau

**DOI:** 10.3390/plants14152325

**Published:** 2025-07-27

**Authors:** Mingxue Xiang, Gang Fu, Junxi Wu, Yunqiao Ma, Tao Ma, Kai Zheng, Zhaoqi Wang, Xinquan Zhao

**Affiliations:** 1 State Key Laboratory of Plateau Ecology and Agriculture, Qinghai University, Xining 810018, China2023990054@qhu.edu.cn (Y.M.); 2023990036@qhu.edu.cn (T.M.); zhengkai2019@qhu.edu.cn (K.Z.); xqzhao@nwipb.cas.cn (X.Z.); 2 Lhasa Plateau Ecosystem Research Station, Key Laboratory of Ecosystem Network Observation and Modeling, Institute of Geographic Sciences and Natural Resources Research, Chinese Academy of Sciences, Beijing 100101, China; fugang@igsnrr.ac.cn (G.F.); wujx@igsnrr.ac.cn (J.W.); 3 Qinghai Sanjiangyuan Grassland Ecosystem National Observation and Research Station, Qinghai University, Xining 810018, China

**Keywords:** alpine grasslands, biomass trade-off, precipitation, Sanjiangyuan, Qinghai–Tibet Plateau

## Abstract

Climate change alters plant biomass allocation and aboveground–belowground trade-offs in grassland ecosystems, potentially affecting critical functions such as carbon sequestration. However, uncertainties persist regarding how precipitation gradients regulate (1) responses of aboveground biomass (AGB), belowground biomass (BGB), and total biomass in alpine grasslands, and (2) precipitation-mediated AGB-BGB allocation strategies. To address this, we conducted a large-scale field survey across precipitation gradients (400–700 mm/y) in the Sanjiangyuan alpine grasslands, Qinghai–Tibet Plateau. During the 2024 growing season, a total of 63 sites (including 189 plots and 945 quadrats) were sampled along five aridity classes: <400, 400–500, 500–600, 600–700, and >700 mm/y. Our findings revealed precipitation as the dominant driver of biomass dynamics: AGB exhibited equal growth rates relative to BGB within the 600–700 mm/y range, but accelerated under drier/wetter conditions. This suggests preferential allocation to aboveground parts under most precipitation regimes. Precipitation explained 31.71% of AGB–BGB trade-off variance (random forest IncMSE), surpassing contributions from AGB (17.61%), specific leaf area (SLA, 13.87%), and BGB (12.91%). Structural equation modeling confirmed precipitation’s positive effects on SLA (β = 0.28, *p* < 0.05), AGB (β = 0.53, *p* < 0.05), and BGB (β = 0.60, *p* < 0.05), with AGB-mediated cascades (β = 0.33, *p* < 0.05) dominating trade-off regulation. These results advance our understanding of mechanistic drivers governing allometric AGB–BGB relationships across climatic gradients in alpine ecosystems of the Sanjiangyuan Region on the Qinghai–Tibet Plateau.

## 1. Introduction

The trade-off between aboveground biomass (AGB) and belowground biomass (BGB), which arises from the allocation of limited resources to cope with environmental pressures, serves as a crucial diagnostic indicator of plant physiological strategies [1,2,3]. This partitioning reflects photosynthetic investment priorities across shoot and root organs [4] and arises from differential selection pressures under heterogeneous environmental conditions [3]. As a cornerstone of plant life–history trade-offs, biomass allocation patterns directly influence niche partitioning and longevity [2,5]. Two frameworks dominate explanatory paradigms: (1) the Optimal Partitioning Hypothesis [6], which posits adaptive biomass adjustments to maximize resource acquisition efficiency, and (2) the Isometric Allocation Hypothesis [7], proposing universal allometric scaling laws across taxa [2,3,8]. Empirical studies present conflicting perspectives: while some emphasize environmental determinism (climate, soil nutrients, and land use), others argue for intrinsic metabolic or phylogenetic constraints overriding external drivers [2,3,8,9,10,11]. However, the spatial distribution of plant biomass (AGB, BGB, and total biomass) along climatic gradients, as well as the responses of AGB–BGB trade-offs to these gradients in the Sanjiangyuan Region of the Qinghai–Tibet Plateau, remain uncertain.

The Sanjiangyuan Region, a critical hydrological nexus encompassing the headwaters of the Yangtze, Yellow, and Mekong Rivers, anchors the ecological security of the Qinghai–Tibet Plateau [12]. This high-altitude landscape (3500–4800 m *a.s.l*.) sustains one of China’s most biodiverse alpine ecosystems, supporting 385 vascular plant species from 36 families, with foundation species including *Kobresia pygmaea*-dominated meadows (70–90% cover) and *Stipa purpurea*-*Carex moorcroftii* steppe communities [13]. These alpine grasslands represent a fragile ecosystem with heightened ecological sensitivity to global environmental changes and anthropogenic pressures [14], characterized by Mattic Gelic Cambisols soils (pH 7.2–8.1; organic carbon 2.1–4.8%) that support distinct biomass allocation strategies across precipitation gradients [13]. Recent decades have seen extensive research on grassland productivity and biomass dynamics [15,16,17,18], but some nondeterminisms persist in the distribution of plant biomass and biomass trade-off between aboveground and belowground elements. Some researchers suggested that climate change, especially the increase in precipitation, is regarded as the main driving factor for grassland productivity in the Sanjiangyuan Region [15], whereas others emphasize temperature-mediated controls on biomass accrual [16]. However, through the application of remote sensing and spatial statistical analysis methods, Li et al. [17] demonstrated that natural factors, including temperature and precipitation, play a significant role in driving ecosystem changes in this region. Therefore, the question of whether precipitation or temperature exerts a dominant influence on plant biomass in the Sanjiangyuan Region remains controversial.

Despite extensive research efforts spanning climate–biomass linkages [16], some uncertainties still persist. Notably, BGB dynamics and their climatic trade-offs with AGB remain underexplored, a significant oversight given BGB’s disproportionate role in alpine carbon sequestration [19]. Resolving these uncertainties requires integrating large-scale field sampling with long-term monitoring to unravel climate–gradient-driven allocation mechanisms. Such advances would provide actionable insights for safeguarding this climate-vulnerable ecosystem amidst accelerating global change. Here, we conducted a large-scale field campaign across precipitation gradients (<400, 400–500, 500–600, 600–700, and >700 mm/y) in the Sanjiangyuan alpine grasslands of the Qinghai–Tibet Plateau, sampling plant biomass components (AGB, BGB, and total biomass) to test predictions of the two partitioning theories. This framework posits that plants dynamically adjust biomass allocation among organs to maximize resource acquisition efficiency [6]. Guided by this, we hypothesized that optimal allocation theory would predominantly govern biomass partitioning patterns between plant organs across the precipitation gradients. Our study objectives were to (1) quantify spatial distributions of plant biomass pools (AGB, BGB, and total) along precipitation gradients, and (2) assess precipitation drivers governing AGB–BGB allometric scaling relationships across the Sanjiangyuan Region. By integrating gradient analyses with allocation theory, these insights advance the mechanistic understanding of how alpine plants optimize resource use in response to environmental changes.

## 2. Results

### 2.1. Distributions of Plant Biomass

**Figure 1:**
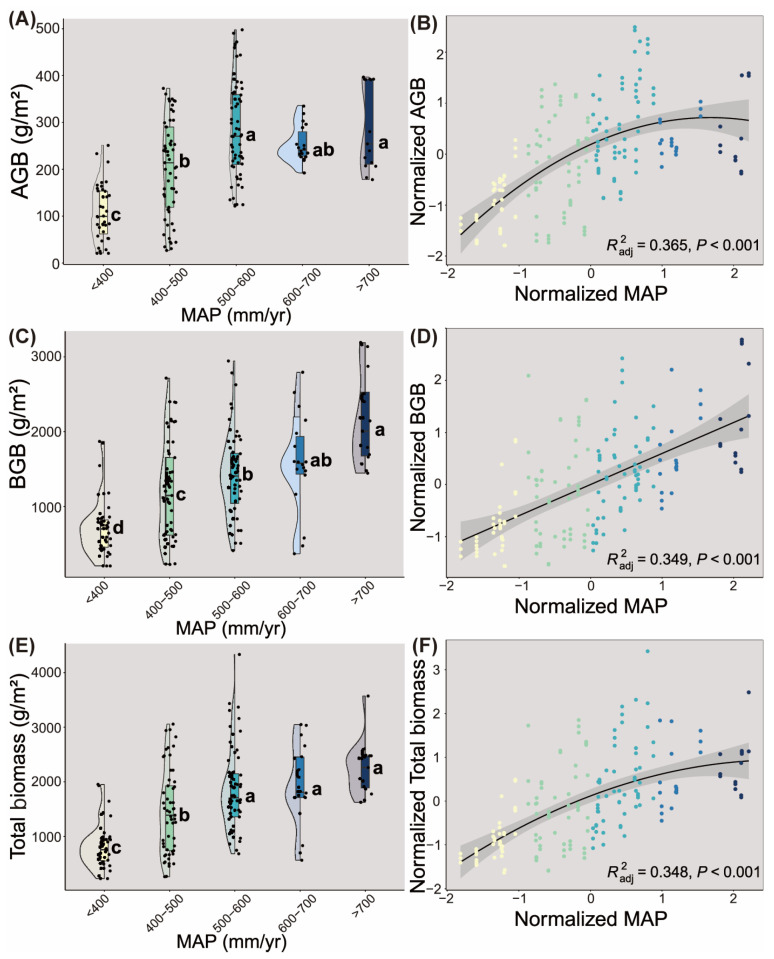
Patterns of plant aboveground biomass (AGB, (**A**,**B**)), belowground biomass (BGB, (**C**,**D**)), and total biomass (**E**,**F**) across gradients of mean annual precipitation or along the mean annual precipitation in the Sanjiangyuan Region of Qinghai–Tibet Plateau. Lowercase letters (a, b, c, and d) signify differences in AGB, BGB, and total biomass (*p* < 0.05) across gradients of mean annual precipitation.

In the alpine grasslands of the Sanjiangyuan Region on the Qinghai–Tibet Plateau, across the precipitation gradient, we observed distinct shifts in plant community composition. Species richness ranged from 1 to 12 taxa per quadrat, with maximum diversity occurring in intermediate precipitation zones (400–500 mm/y: 3–11 species) compared to arid (<400 mm: 1–12) and humid (>700 mm: 3–7) extremes. In addition, AGB, BGB, and total biomass were significantly influenced by precipitation (Figure 1). Specifically, these biomass components exhibited positive correlations with increasing annual precipitation (Figure 1B,D,F). Below 700 mm/y precipitation, marked variations in BGB and total biomass were observed across precipitation gradients. Notably, AGB showed significant differentiation across precipitation gradients at lower thresholds below 600 mm/y (Figure 1A,C,E).

### 2.2. Trade-Off Between Above- and Belowground Biomass

**Figure 2:**
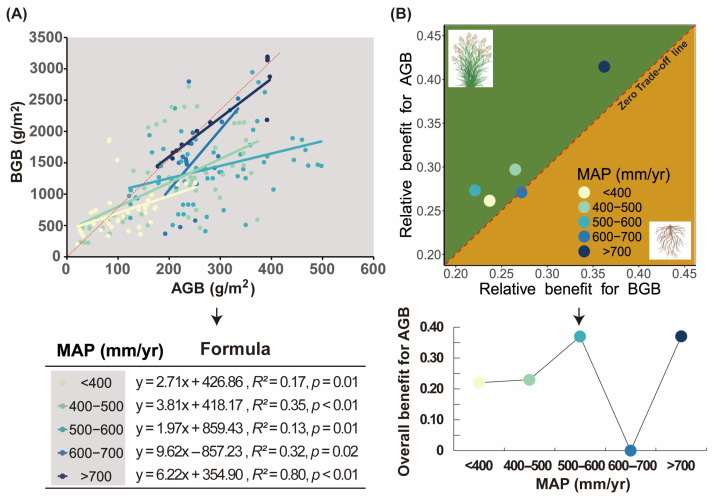
Relationships between plant aboveground and belowground biomass (Panel (**A**)) and their trade-off dynamics (Panel (**B**)) across mean annual precipitation gradients in the Sanjiangyuan Region of Qinghai–Tibet Plateau. The red dotted lines in Panels (**A**,**B**) indicate the 1:1 reference line and zero-trade-off line, respectively. The zero-trade-off baseline demarcates the threshold where no biomass allocation bias occurs. Data points positioned above this baseline reflect preferential aboveground biomass allocation, whereas those below indicate belowground allocation predominance. The vertical distance from individual points to the zero-trade-off baseline quantitatively corresponds to the trade-off intensity.

Precipitation exerted differential effects on the trade-off between AGB and BGB (Figure 2). A distinct allometric growth relationship was observed between these biomass compartments (Figure 2A). Notably, AGB exhibited slower growth rates than BGB, specifically within the 600–700 mm/y precipitation range, while demonstrating faster growth across other precipitation gradients. This pattern corresponds to a biomass allocation priority to aboveground components in most rainfall conditions (Figure 2B). It is worth noting that the 600–700 mm/y precipitation range exhibited an absence of significant trade-off dynamics between AGB and BGB allocation (Figure 2B).

### 2.3. Influencing Factors of Biomass Trade-Off

**Figure 3:**
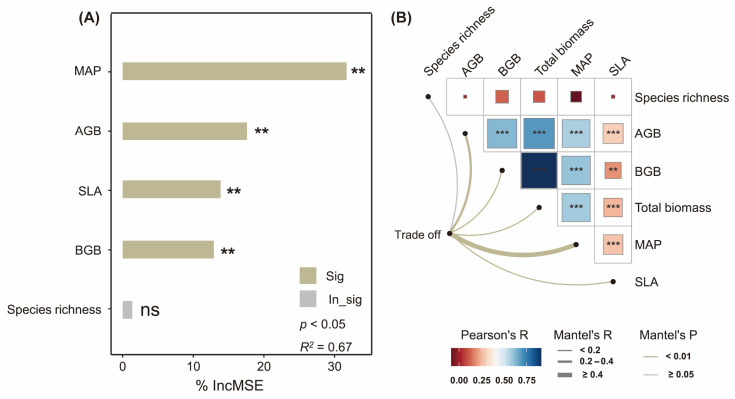
Pearson correlation between plant biomass trade-off and plant species diversity, aboveground biomass (AGB), belowground biomass (BGB), total biomass, mean annual precipitation (MAP), and plant-specific leaf area (SLA, Panel (**A**)). The identification via random forest analysis of MAP, AGB, SLA, BGB, and plant species diversity as dominant factors in trade-off changes (Panel (**B**)). ** *p* < 0.01; *** *p* < 0.001.

**Figure 4:**
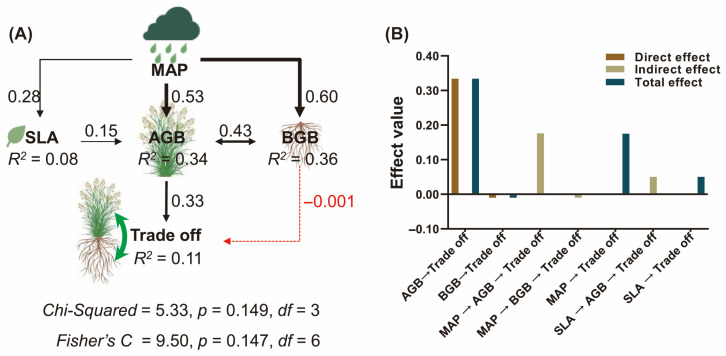
Dependencies of plant biomass trade-off on mean annual precipitation (MAP), aboveground biomass (AGB), specific leaf area (SLA), and belowground biomass (BGB). Black solid lines represent significantly positive relationships, while the red dotted line indicates insignificant relationships (Panel (**A**)). Quantification of standardized factor effects appears (Panel (**B**)).

Biomass characteristics (including AGB, BGB, and specific leaf area) and precipitation significantly influenced biomass trade-offs, whereas plant diversity (e.g., species richness) showed no detectable effects (Figure 3A,B). Specifically, precipitation emerged as the dominant contributor to biomass allocation trade-offs (IncMSE = 31.71%), followed sequentially by AGB (17.61%), specific leaf area (13.87%), and BGB (12.91%, Figure 4A). Pathways analysis revealed that while precipitation enhanced the specific leaf area, aboveground biomass, and belowground biomass (all *p* < 0.01), its influence on biomass trade-offs was primarily mediated through aboveground biomass allocation pathways (standardized coefficient: β = 0.33, Figure 4).

## 3. Discussion

### 3.1. The Effects of Climate and Plant Characteristics on Above- and Belowground Biomass

**Figure A1:**
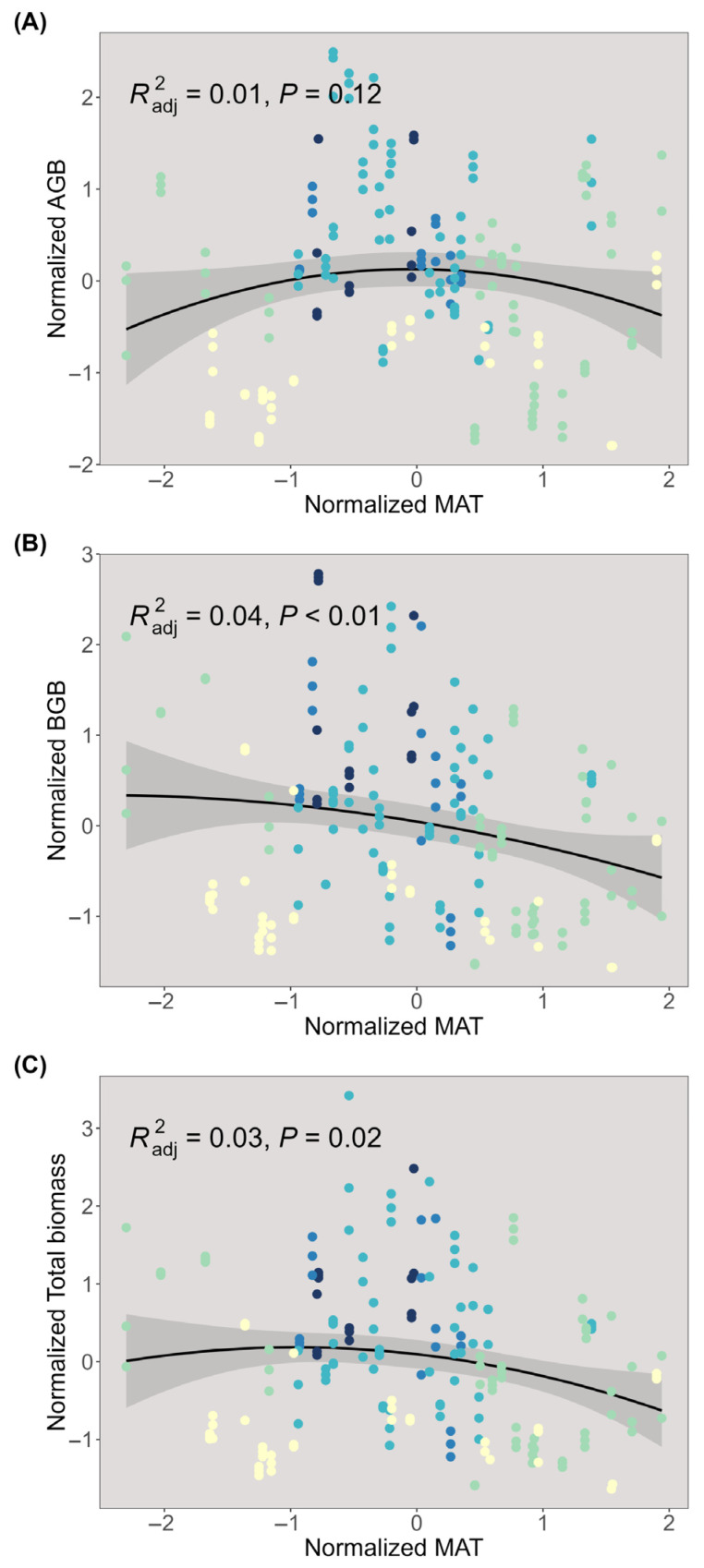
Patterns of plant aboveground biomass (AGB, (**A**)), belowground biomass (BGB, (**B**)), and total biomass (**C**) along the mean annual temperature in the Sanjiangyuan Region of QTP.

Precipitation plays a critical role in shaping the distribution of plant biomass across the Sanjiangyuan Region of the Qinghai–Tibet Plateau. First, our analyses demonstrated that plant biomass components—including AGB, BGB, and total biomass—exhibited significant positive correlations with mean annual precipitation (Figure 1). This aligns with documented precipitation-driven biomass dynamics in alpine grasslands of the northern/central Tibetan Plateau [20,21] and broader Eurasian temperate grasslands [22]. The consistency across these studies reinforces water availability as the dominant limiting factor for biomass accumulation in xeric grassland ecosystems. Second, temperature exhibited weaker regulatory effects on biomass distribution patterns. A weak unimodal relationship emerged between AGB and the mean annual temperature (Figure A1), suggesting minimal thermal constraints on aboveground productivity. BGB displayed a subtle negative correlation with temperature (Figure A1). Notably, this decoupled temperature response between AGB and BGB mirrors findings in the alpine meadows of the eastern QTP [23]. Collectively, these results underscore precipitation as the primary climatic driver of plant biomass responses to climate change in the Sanjiangyuan Region, with temperature playing a secondary or indirect role.

**Figure A2:**
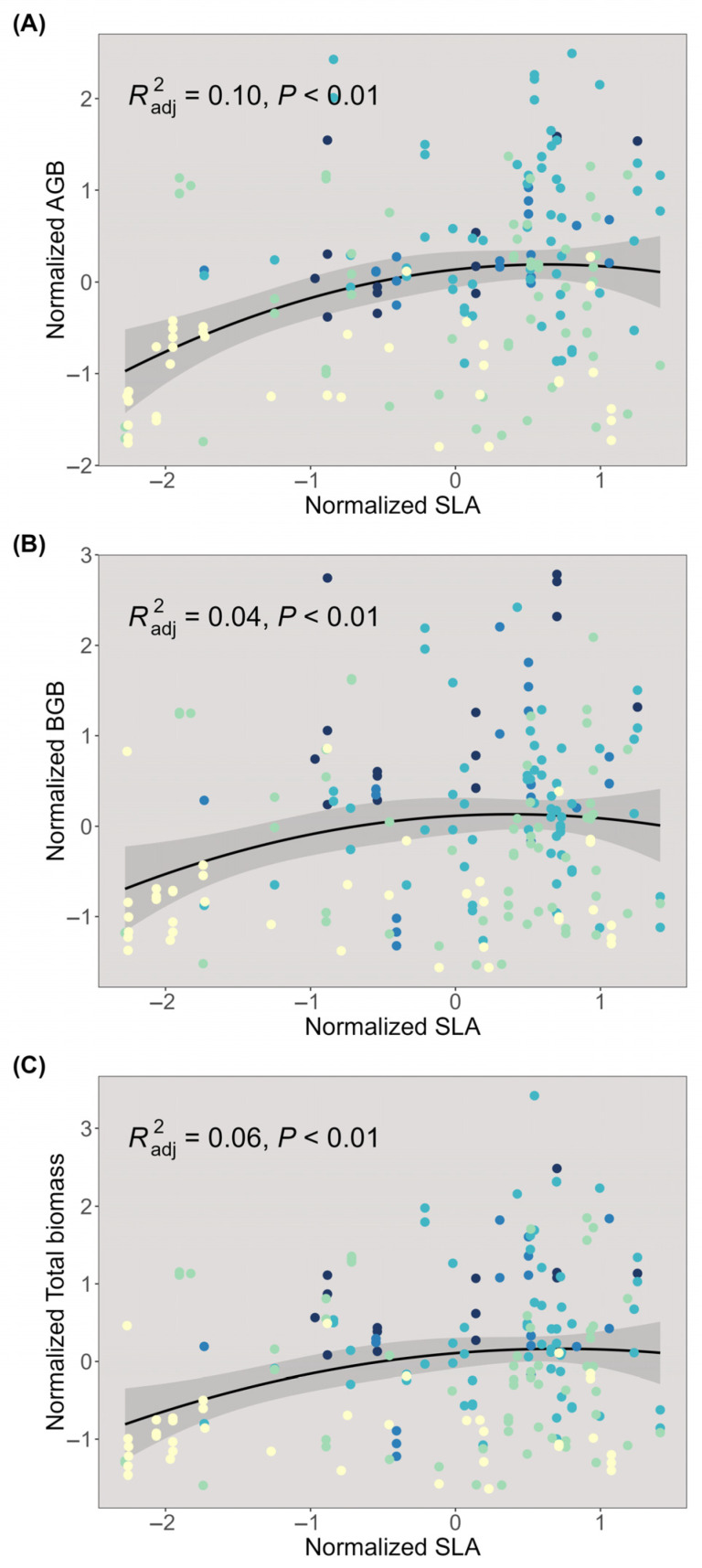
Relationships between normalized specific leaf area (SLA) and normalized aboveground biomass (AGB, (**A**)) and normalized belowground biomass (BGB, (**B**)) and normalized total biomass (**C**) in the Sanjiangyuan Region of QTP.

**Figure A3:**
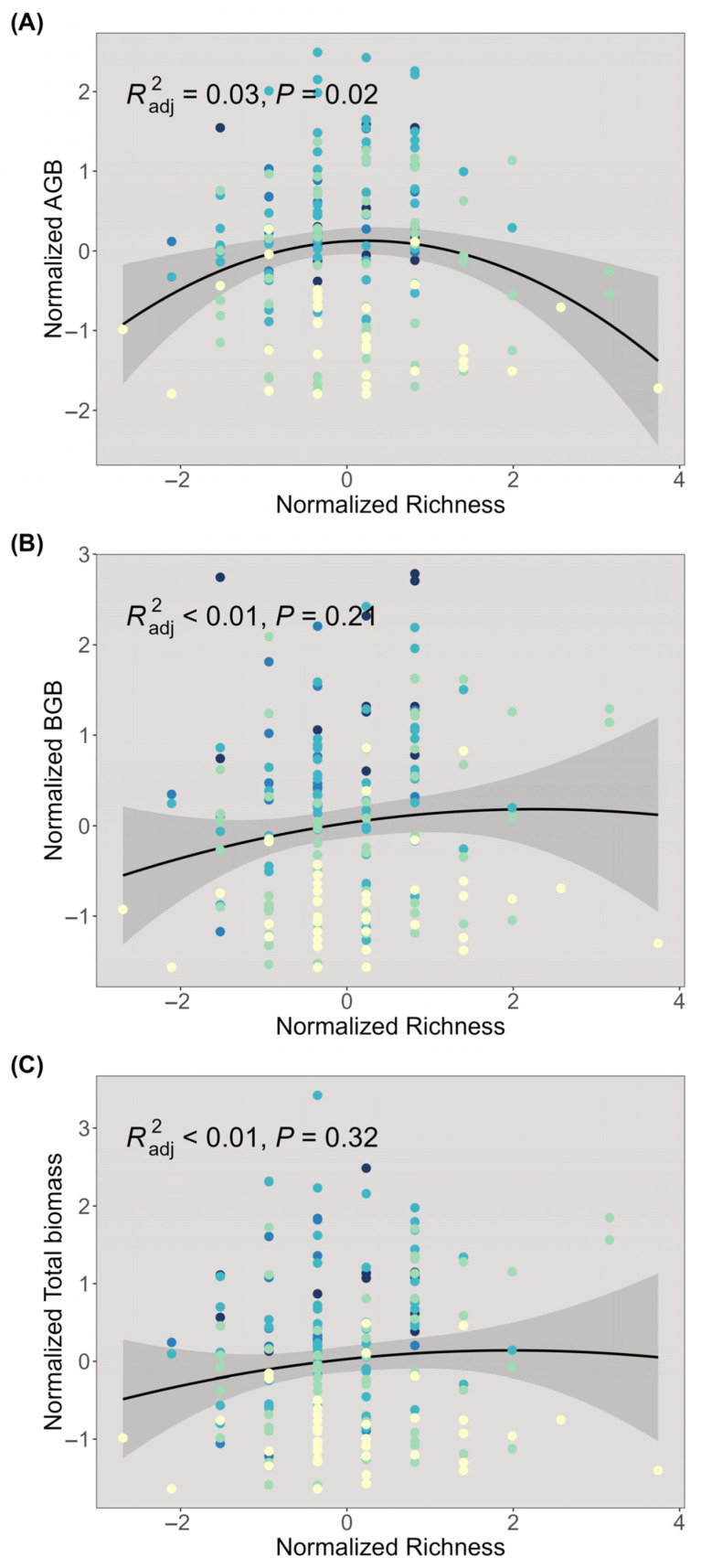
Relationships between normalized species richness and normalized aboveground biomass (AGB, (**A**)) and normalized belowground biomass (BGB, (**B**)) and normalized total biomass (**C**) in the Sanjiangyuan Region of QTP.

Plant functional traits and community composition are jointly associated with plant biomass in the alpine grasslands of Sanjiangyuan, with species richness exhibiting differential above- versus belowground linkages. First, our analyses revealed a weak unimodal relationship between SLA and biomass components (AGB, BGB, and total biomass, Figure A2), contrasting with widely reported linear SLA–biomass correlations in homogeneous communities [24]. This divergence likely stems from the Sanjiangyuan Region’s heterogeneous vegetation mosaics—where distinct plant communities (e.g., sedge- vs. forb-dominated assemblages) exhibit opposing SLA–climate response trajectories [13,25]. Notably, SLA’s ecological plasticity varies substantially across codominant species, as evidenced by *Kobresia pygmaea* (low SLA, high biomass stability) and *Oxytropis glacialis* (high SLA, climate-sensitive biomass) under identical precipitation regimes [26,27]. Second, species richness displayed context-dependent biomass associations: a hump-shaped relationship with AGB (Figure A3), aligning with biodiversity productivity theory [13,20], but decoupled linkages to BGB and total biomass (Figure A3). This belowground decoupling mirrors methodological constraints in partitioning species-specific root contributions within mixed communities [20]—a limitation exacerbated by our root sampling protocol. By employing the ring knife method, we captured legacy root systems from prior growing seasons alongside current-year roots, thereby diluting richness–BGB signal fidelity. Collectively, these findings redefine trait-based biomass allocation models by emphasizing the following: (1) community context overrides leaf economics in heterogeneous alpine systems, and (2) methodological biases inherent in belowground productivity assessments.

### 3.2. Precipitation-Driven Aboveground Biomass Dominance Mediates Alpine Grassland Allocation Trade-Offs

Precipitation drives dynamic trade-offs in AGB–BGB partitioning in the Sanjiangyuan Region of the Qinghai–Tibet Plateau. First, our data revealed spatially heterogeneous allometric scaling relationships between AGB and BGB across precipitation gradients (Figure 2A), with these allocation patterns directly governing biomass trade-off dynamics (Figure 2B). This supports our hypothesis that precipitation mediates optimal biomass allocation strategies. While isometric AGB–BGB relationships dominate in alpine meadows and steppes when climatic conditions are not considered [8], divergent responses emerge when precipitation variability is considered—a pattern corroborated by global grassland studies [28,29]. Second, we observed threshold-dependent allocation shifts along the precipitation gradient. Below the 600 mm/y threshold, AGB prioritization dominated in our system (Figure 2A,B), aligning with Li et al.’s [30] framework of water-mediated allocation trade-offs. However, we acknowledge contrasting findings in extremely arid ecosystems (<300 mm/y), where BGB dominance often occurs to enhance water acquisition [31,32]. In our study region, the persistence of AGB investment at intermediate drought levels (400–600 mm) may reflect: (a) The shallow rooting depth (30–50 cm) of dominant *Kobresia* species [33]. (b) Competitive strategies for light capture in dense turf communities [34]. (c) Evolutionary adaptations to combined water and cold stress [35]. However, nonlinear responses emerge beyond this threshold, as excessive moisture disrupts predictable allocation patterns—a phenomenon attributed to oxygen limitation in waterlogged soils [36].

Notably, the observed zero trade-off between AGB and BGB within the 600–700 mm/y precipitation range (Figure 2B) likely reflects compensatory allocation strategies under optimal hydrological conditions. Previous studies demonstrate that moderate precipitation surpluses alleviate both water and nutrient constraints [37,38], enabling simultaneous investment in photosynthetic tissues (AGB) and root systems (BGB) without competitive trade-offs [2,39]. However, above 700 mm/y, the resurgence of AGB dominance over BGB coincides with water saturation effects. Prolonged soil waterlogging beyond this threshold restricts root respiration and nitrogen uptake efficiency due to hypoxic conditions [40], forcing plants to prioritize carbon allocation to aboveground organs, a survival strategy documented in water-saturated alpine wetlands [41]. Therefore, these findings position precipitation as both a primary driver and a nonlinear modulator of biomass partitioning trade-offs in high-altitude grasslands.

Precipitation dominates hierarchical controls over biomass allocation trade-offs in the Sanjiangyuan alpine grasslands, with aboveground biomass serving as the pivotal mediator. Our integrated random forest and mantel analyses revealed that the mean annual precipitation exerted the strongest relative influence on AGB–BGB trade-off (31.71%, *p* < 0.01), followed by AGB (17.61%, *p* < 0.05), SLA (13.87%, *p* < 0.05), and BGB (12.91%, *p* < 0.05, Figure 3). This hierarchy aligns with precipitation as the master regulator of biomass partitioning in water–heat-limited ecosystems [8,42,43], while SLA’s subordinate role aligns with trait-mediated carbon reallocation mechanisms in drought-adapted grasslands [44]. These findings underscore precipitation’s nonlinear capacity to override intrinsic plant traits in governing allocation trade-offs under aridification pressures.

Structural equation modeling revealed statistically supported pathways consistent with hypothesized precipitation effects, though we emphasize that these represent robust associations rather than demonstrated causal relationships (Figure 4). Significant positive effects of precipitation on AGB (β = 0.53, *p* < 0.05) and BGB (β = 0.60, *p* < 0.05) were observed, with SLA showing marginal responsiveness (β = 0.28, *p* < 0.05). Precipitation’s indirect control over AGB–BGB trade-offs operated predominantly via AGB-driven canopy hydraulic efficiency (β = 0.33, *p* < 0.05), a pattern consistent with xylem–phloem coordination models in herbaceous plants [45]. SLA’s weak mediation (β = 0.28, *p* < 0.05) deviates from leaf economic spectrum tenets [46], likely reflecting QTP-specific constraints: low temperatures suppress SLA-linked photosynthetic returns, favoring root carbon storage over leaf area expansion. By positioning AGB as a hydraulic relay—a functional bottleneck through which precipitation modulates whole-plant carbon routing—our findings extend Optimal Partitioning Theory to water-limited alpine systems [6].

### 3.3. Uncertainties in Plant AGB–BGB Trade-Offs

Firstly, annual precipitation and its seasonal distribution regulate plant resource allocation priorities between photosynthetic organs (leaves) and absorptive organs (roots) by modulating soil water availability [47,48,49]. While drought stress typically drives biomass partitioning toward belowground components to enhance water acquisition (e.g., via root deepening) [44,50], our study revealed a counterintuitive pattern: AGB–BGB trade-offs consistently favored aboveground allocation across all precipitation gradients (Figure 2B). This suggests that soil water availability may not be a critical limiting factor in the Sanjiangyuan Region (Figure 3 and Figure 4), despite precipitation’s overarching influence on allocation dynamics.

Secondly, while our study provides novel insights into precipitation-driven biomass allocation dynamics, we acknowledge methodological constraints that warrant discussion. The use of county-level meteorological station data (2001–2022 averages) introduces potential uncertainties when extrapolated to plot-scale sampling points (Figure 2). Although we applied rigorous spatial interpolation techniques (e.g., Kriging with elevation correction), the limited density of high-altitude stations (<15 stations across 250,000 km^2^) may inadequately capture microclimatic variations critical to alpine grassland responses. This scale mismatch between climate drivers (long-term averages) and biomass measurements (single-year 2024 data) could decouple observed allocation patterns from interannual climate variability, a recognized limitation in gradient studies [51]. The single-year biomass sampling (2024) inherently overlooks interannual dynamics, particularly for AGB, which exhibits 20–40% annual fluctuations in alpine systems [52]. While our precipitation gradient approach partially compensates for temporal limitations through space-for-time substitution [53], validation against long-term station data (e.g., Sanjiangyuan Alpine Wetland Ecosystem Observation Station’s 15-year biomass records) would strengthen temporal generalizability. Future studies should integrate multi-year field campaigns across drought/wet extremes to test allocation consistency. Future research could leverage satellite-derived biomass proxies (e.g., MODIS NDVI phenology composites) calibrated with ground measurements to enhance AGB monitoring [54]. Promising opportunities include (1) coupling plot-level BGB measurements with Sentinel-2-based AGB estimates (30 m resolution) [54] to validate spatial allocation patterns across unsampled areas; (2) reconstructing decadal biomass trends using Landsat archives (1984–present); and (3) refining climate-allocation models through integrated ground-truth and remote sensing approaches. These directions offer tractable pathways to advance our understanding of biomass allocation dynamics in alpine ecosystems.

Thirdly, community composition and interspecific competition likely override abiotic constraints in shaping grassland biomass allocation strategies [11,55]. Future research should prioritize disentangling the interactive effects of grassland types, plant community species assemblages, and interspecific competition in AGB–BGB trade-offs. Contrary to expectations that low-temperature environments (e.g., alpine grasslands) would prioritize investment in belowground storage organs (e.g., rhizomes) due to shortened growing seasons [56], our analyses detected minimal temperature control over AGB–BGB partitioning (Figure A1). This paradox may reflect compensatory mechanisms such as light competition, where canopy space preemption drives AGB prioritization under high irradiance [57,58]. Consequently, light-use efficiency and photoadaptive strategies warrant targeted investigation in alpine grassland allocation models.

Lastly, although large-scale soil nutrient measurements were logistically constrained in our Sanjiangyuan research efforts due to prohibitive testing costs and logistical constraints, emerging evidence suggests that soil nutrient availability may modulate AGB–BGB trade-off slopes [2,39]. Future studies must address the synergistic effects of climate change and edaphic heterogeneity on biomass partitioning, particularly in carbon-sensitive alpine ecosystems. While grazing (intensity) undeniably modulates plant biomass partitioning [9], our study design intentionally homogenized this variable to isolate climatic drivers: (1) All sampling sites are situated within year-round grazed alpine grasslands under traditional pastoral management. This uniform grazing pressure likely created a background “filter” that standardizes grazing effects across the climatic gradient. While minor local variations exist, they are negligible compared to the pronounced precipitation-driven variations (Figure 1). (2) At the regional gradient scale (~400–700 mm/y), climatic drivers dominated biomass allocation variance, consistent with scaling theory [52]. This hierarchy arises because large-scale precipitation gradients override fine-scale grazing effects. (3) We minimized grazing-induced noise by excluding plots within 500 m of settlements/winter pastures (intensive grazing zones). To fully disentangle grazing–climate interactions, we propose a follow-up study utilizing controlled grazing exclosures along the same precipitation gradient—for instance, leveraging long-term grazing manipulation platforms within the Three-River-Source National Park.

## 4. Materials and Methods

### 4.1. Study Site

**Figure 5:**
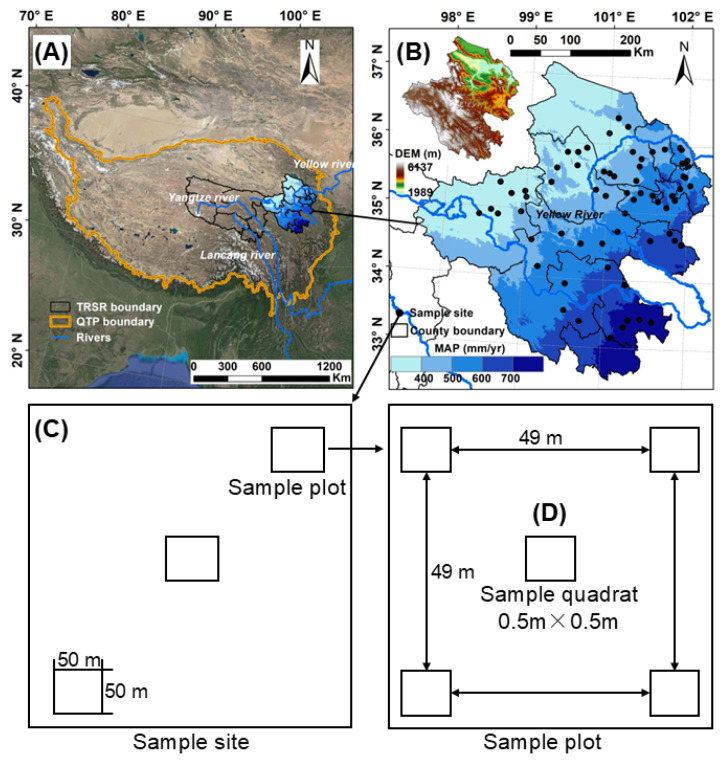
The distribution of plant samples across gradients of mean annual precipitation in the Sanjiangyuan Region of Qinghai–Tibet Plateau. (**A**) Position of the Sanjiangyuan Region in the Qinghai–Tibet Plateau; (**B**) Sampling sites in the Sanjiangyuan Region; (**C**) Sample site with three 50 m × 50 m sample plots; (**D**) Sample plot with five 0.5 m × 0.5 m sample quadrats.

The Sanjiangyuan Region (SR, 31–37° N, 89–104° E, Figure 5A), encompassing 363,000 km^2^ of the Qinghai–Tibet Plateau, functions as Asia’s pivotal water tower. Its alpine–periglacial ecosystems generate 25–49% of discharge in three transboundary rivers (Yangtze, Yellow, and Mekong), sustaining downstream agricultural and urban systems [59]. Vertical climatic stratification (3500–6564 m *a.s.l.*) creates temperature (ranging from −5.6 °C to 3.8 °C) and precipitation (262–772 mm/y) gradients that structure endemic biodiversity hotspots [60]. Hydrological monitoring since 1961 has confirmed SR’s role as a climate-sensitive buffer against downstream hydrological extremes.

### 4.2. Climate Data Processing

The mean annual temperature (MAT) and mean annual precipitation (MAP) for 2001–2022 were derived using Universal Kriging, where elevation was included as a covariate in the trend component to correct for orographic effects. This approach aligns with standard practices in mountainous regions, where elevation-driven temperature lapse rates and precipitation gradients necessitate covariate-based interpolation.

### 4.3. Plant Sampling

Plants were sampled in August 2024, coinciding with peak coverage and aboveground biomass for most species. A total of 63 sampling sites for plant sampling were selected (Figure 5B), with three 50 m × 50 m sampling plots per site (Figure 5C) and five 0.5 m × 0.5 m quadrats per plot (Figure 5D), yielding 945 quadrat-level observations. Quadrats were systematically positioned to assess plant community species diversity, canopy height, and specific leaf area (SLA). All vascular plants within each quadrat were clipped at ground level and processed following the standard protocols [61]. Samples were oven-dried at 65 °C to constant mass (72 h minimum) and were weighed to quantify the aboveground biomass. Belowground biomass was assessed using a 5 cm-diameter soil core to a 30 cm depth, with root samples rinsed, dried (65 °C, 48 h), and weighed following Cornelissen et al. [62].

### 4.4. Data Calculation

#### 4.4.1. Statistic Analysis of Plant Biomass

Plant biomass patterns were analyzed using assumption-driven statistical protocols. Statistical assumptions were rigorously evaluated: *Shapiro–Wilk tests* confirmed non-Gaussian distributions for AGB (all *p* < 0.05), and *Levene’s tests* indicated heteroscedasticity (*p* < 0.05). These findings justified our use of non-parametric *Kruskal–Wallis H-tests* with *Dunn’s* post hoc corrections for group comparisons [63]. Parametrically distributed BGB and total biomass underwent *ANOVA* followed by *Tukey’s HSD* (α = 0.05). In addition, the relationships between AGB and BGB, as well as between AGB, BGB, and total biomass and climate factors were modeled using generalized additive models.

#### 4.4.2. Calculation of the Biomass Trade-Off

Firstly, we calculated the relative benefit of AGB/BGB (*RB*, Equation (1)), as described below:

(1) $$RB=\frac{x_{i}-x_{min}}{x_{max}-x_{min}}$$

where

- *x_i_* = observed AGB (g/m^2^) or BGB (g/m^2^) in the *i*-th sampling quadrat;
- *x_min_* and *x_max_* = minimum and maximum observed values of AGB or BGB (g/m^2^) across all quadrats, respectively.

To quantify the biomass allocation trade-off, we calculated the root mean square error (*RMSE*, Equation (2)) between the observed and expected biomass ratios [9,10]. The RMSE was normalized by plot size to enable cross-comparisons. This metric captures both the magnitude of deviation from balanced allocation (*RB*_AGB_/*RB*_BGB_ = 1) and the systematic bias toward above- or belowground allocation.

(2) $$RMSE=\sqrt{\frac{1}{n-1}\sum_{i=1}^{n}\;(RB-\bar{B}{)}^{2}}$$

where

- $\bar{B}$ is the expected biomass benefit (mean of all AGB or BGB measurements);
- *n* = total number of quadrats.

*RMSE* values were interpreted as follows:

- Positive values (*RB*_AGB_/*RB*_BGB_ > 1): AGB allocation dominance;
- Negative values (*RB*_AGB_/*RB*_BGB_ < 1): BGB prioritization;
- Values approaching zero: Balanced allocation.

#### 4.4.3. Mantel Test, Random Forest Analysis, and Structural Equation Model

Multivariate analyses were conducted using R (v4.4.0) [64]. We first employed the ‘*linkET*’ package in R to conduct correlation analyses between biomass allocation trade-offs and key drivers: MAP, species diversity (species richness), AGB, BGB, total biomass, and specific leaf area (SLA). We implemented random forest analysis with %IncMSE calculation. Variable importance was confirmed via conditional permutation tests [65]. Based on these results, we developed a priori structural equation models (SEMs) using ‘*piecewiseSEM*’ [66]. Prior to predictive modeling, we established causal relationships using Directed Acyclic Graphs (DAGs) incorporating known ecological mechanisms; SEM with explicit causal pathways; and sensitivity analyses for potential unmeasured confounding. Only after confirming these causal relationships did, we proceed with predictive modeling using random forest. Based on established causal relationships, the SEM evaluated the hypothesized pathways through which the MAP influences SLA, AGB, BGB, and their trade-offs. We employed the *χ^2^* test (*p* > 0.05) for overall model specification, *RMSEA* (<0.08) and *CFI* (> 0.90) for approximate fit, and Fisher’s C statistic for model parsimony. This causal framework preceded and informed subsequent predictive analyses [66].

## 5. Conclusions

This study investigated the partitioning of plant aboveground (AGB) and belowground biomass (BGB) and their relationships with biotic–abiotic drivers in the alpine grasslands of the Sanjiangyuan Region, Qinghai–Tibet Plateau. Precipitation emerged as the critical determinant of biomass distribution patterns across the region. Moreover, it dynamically regulates AGB–BGB trade-offs through threshold-dependent mechanisms: AGB prioritization dominated under two distinct precipitation regimes (<600 mm/y and >700 mm/y), whereas balanced allocation occurred at intermediate levels (600–700 mm/y). Structural analyses revealed precipitation’s dominant hierarchical control over allocation trade-offs, with AGB serving as the pivotal mediator. Crucially, while existing studies emphasize drought-driven BGB prioritization in water-limited ecosystems, our findings demonstrate persistent AGB dominance in Sanjiangyuan grasslands, a paradigm shift challenging deep-rooted water limitation assumptions. Future research should prioritize elucidating the hierarchy of temperature–light–soil nutrient interactions modulating biomass allocation under precipitation-dominated regimes, particularly through manipulative experiments across aridity gradients.

## Data Availability

All the required data are uploaded as Supplementary Materials.

## Supplementary Materials

The following supporting information can be downloaded at: https://www.mdpi.com/article/10.3390/plants14152325/s1.
